# High expression of UBE2T predicts poor prognosis and survival in multiple myeloma

**DOI:** 10.1038/s41417-018-0070-x

**Published:** 2019-01-09

**Authors:** Weilong Zhang, Ye Zhang, Zuozhen Yang, Xiaoni Liu, Ping Yang, Jing Wang, Kai Hu, Xue He, Xiuru Zhang, Hongmei Jing

**Affiliations:** 1 Department of Hematology, Lymphoma Research Center, Peking University Third Hospital, 100191 Beijing, China; 2 Melbourne School of Population and Global Health, The University of Melbourne, Melbourne, VIC 3010 Australia; 3 Department of Respiratory Medicine, The First Affiliated Hospital of Gannan Medical University, 341000 Ganzhou, China; 4 Department of Pathology, Beijing Tiantan Hospital Affiliated with Capital Medical University, No. 6 Tiantan Xili, 100050 Beijing, China

**Keywords:** Myeloma, Tumour biomarkers

## Abstract

Multiple myeloma (MM) is one of hematological malignancies, characterized by malignant proliferation of plasma cells. Biomarkers play an important role in evaluating the development and prognosis of MM. Ubiquitin-conjugating enzyme E2T (UBE2T) is served to connect with particular E3 ubiquitin ligase to degraded-related substrates, contributing to DNA repair in the Fanconi anemia pathway. Also, numerous evidences reported that UBE2T is closely related to cell proliferation and carcinogenesis. However, the relationship between MM and UBE2T has not been studied. Here, we integrated eight datasets and analyzed the relationship of expression of UBE2T and ISS, 1q21, relapse and survival in MM 2684 patients (totally 2893 samples). We found that the expression of UBE2T increased with the deterioration of MM (*P* = 1.4e-07), especially in the early stage. UBE2T is closely related to IgG serotype MM (*P* = 6.9e-05). High expression of UBE2T is associated with poor survival and prognosis (EFS: *P* = 1.43e-03, OS: *P* = 5.47e-05). UBE2T is likely to play a part in the cell division pathway, affecting the survival and prognosis of MM. Therefore, UBE2T could be considered as an early alternative biomarker for the prognosis of MM.

## Introduction

Multiple myeloma (MM), a plasma cell malignancy, is considered as the second most common blood system malignancy [1, 2]. It derives from the malignant proliferation of monoclonal plasma cell and secretes plenty of monoclonal immunoglobulin, injuring corresponding organs and tissues [3]. At first, no symptoms were observed in MM patients [4]. However, with the development of the disease, anemia, bone pain, and frequently infection were manifested [5]. Majority of MM occurred in elders [6]. To date, chemotherapy and autologous hematopoietic stem-cell transplantation (ASCT) are two the most effective therapies for MM [7]. Although numerous researches have been conducted on MM, the etiology of MM still remain poorly understand. MM is malignant with high recurrence and the median overall survival in patients varies from several months to years [8]. The survival of MM is closely related to the clinical stage of MM [8]. International staging system (ISS) stage criterion is regarded as the “gold standard” for the clinical stage of MM, which define MM into stage ISS I, ISS II and ISS III [9, 10]. According to University of Arkansas for Medical Science (UAMS), MM has seven molecular types: proliferation, MMSET (MS), hyperdiploid, low bone disease (LB), CD1, CD2 and MAF/MAFB (MF) [11]. Different molecular types of MM are related to different prognosis. In recent years, biomarkers play an important role in the prognosis of cancer. Therefore, it is necessary to investigate new biomarkers for MM, assisting in evaluating the diagnosis and prognosis of MM.

Ubiquitin-conjugating enzyme E2T (UBE2T), a typical ubiquitin-conjugating enzyme, is served to connect with particular E3 ubiquitin ligase to degrade related substrates [12]. It was initially reported in a patient who suffered from Fanconi anemia (FA) [13]. The development of FA is associated with the maladjustment of the FA pathway that is important to DNA damage repair [14]. UBE2T is essential for the FA pathway. It also plays a crucial role in cellular developments, for example, signal transduction and cell cycle control [15]. UBE2T, located in 1q32.1, has impact on cell proliferation; numerous studies identified that its overexpression results in a variety of tumorigenesis [16]. For instance, UBE2T promotes breast cancer through inhibiting the expression of BRCA1 [17]. The overexpression of UBE2T triggers the AKT/GSK3β/β-catenin pathway, contributing to the development of nasopharyngeal carcinoma [18]. However, limited studies were conducted to investigate the relationship between UBE2T and MM. The role of UBE2T in MM still remains largely unknown.

In this study, we investigated the expression of UBE2T in 2684 patients who suffered from MM, analyzing the relationship between UBE2T and ISS, 1q21, relapse and survival. Our study suggested that the expression of UBE2T is a bad indicator of MM, relating to poor outcomes.

## Methods

### Data source

In our study, we extracted 2684 MM patients’ gene expression microarrays from Gene Expression Omnibus (GEO) database. In GSE24080, 559 samples from 559 cases [19], we analyzed the association between UBE2T and clinical stage, serotype, molecular type, 1q21 amplification, pathway and survival of MM patients. In GSE24080, MM patients were treated through TT2 (Induction therapy: D(T)-PACE, dexamethasone with or without thalidomide; Maintenance: Thalidomide) and TT3 (Induction therapy: VTD-PACE; Maintenance: Bort-Thal-Dex). In GSE82307, 66 samples from 33 cases [20], we analyzed the expression of UBE2T in patients before and after the relapse. In GSE19554, 38 samples from 19 cases [21], we analyzed the expression of UBE2T before and after the first chemotherapy in patients. In GSE19784, 308 samples from 308 cases [22], we analyzed the expression of UBE2T in nine different molecular types. In GSE83503, 585 samples from 585 cases [23], we analyzed the expression of UBE2T in relapse patients and non-relapse patients. In GSE31161, 937 samples from 780 cases, we analyzed the association between UBE2T expression and relapse. In GSE9782, 264 samples from 264 patients [24], we analyzed the association between the expression of UBE2T and drug treatment response (dexamethasone and bortezomib). In GSE39754, 136 samples from 136 cases [25], we analyzed the association between the expression of UBE2T and VAD and ASCT combined therapeutic response. This study was in conformity to the Declaration of Helsinki. This study was approved by the committee of Peking University Third Hospital.

### Microarray analysis

We employed statistical analysis to investigate significant abnormal expressed genes on every microarray dataset. In all, 8.8372, the expression level of UBE2T, was considered as the cut point. According to this cut point, we divided MM patients into two groups. Patients whose UBE2T level higher than 8.8372 consisted of expression high group. However, UBE2T expression level lower than 8.8372 consisted of expression low group. We analyzed the UBE2T expression profiles in both high and low groups. *P* < 0.05 in unpaired *t*-test analysis and foldchange (FC, log2) >0.8 or <−0.8 was utilized to determine the differential expression of genes.

### Gene Ontology analysis

We utilized default parameters of the DAVID tool to analyze the enrichment of pathway between UBE2T expression high and low groups [26]. The results were ranked by *P*-value (−log10).

### Statistics

We employed R software v3.1.3 (ggplot2 and survminer package) to operate the statistical analysis. Kruskal–Wallis test was used to compare multiple sets of samples. The log-rank test was used for survival analysis. *T*-test and Wilcoxon test were used to compare the mean value of two groups. Anova test was used to compare means of more than two groups.

## Results

### The expression of UBE2T in MM patients between different stages

**Fig. 1:**
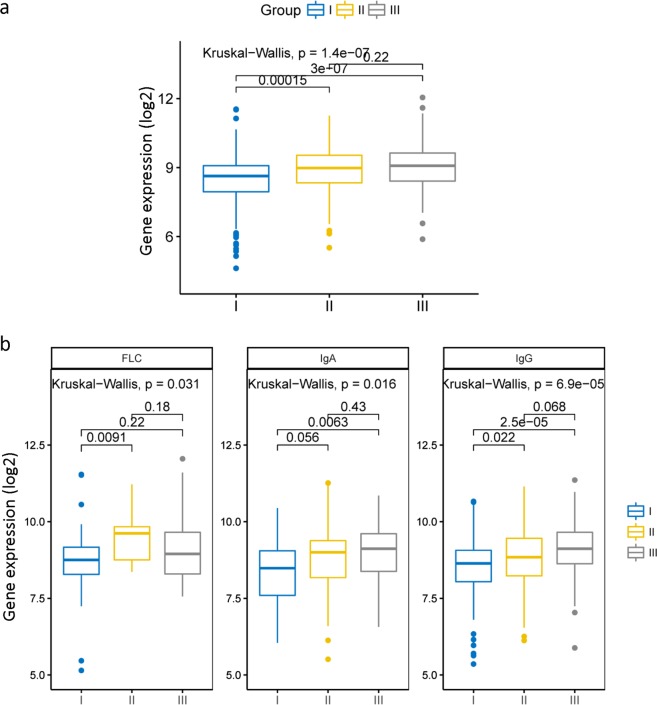
The expression of UBE2T in the different clinical stage of MM. **a** The expression of UBE2T in the different ISS stage of MM. UBE2T expressed highest in stage III patients, while lowest in stage I patients. **b** The expression of UBE2T in three serotypes of under the different ISS stage. FLC serum free light chain, IgA serum immunoglobulin A, IgG, serum immunoglobulin G. Kruskal–Wallis test

In order to understand the expression of UBE2T in MM patients in different stages, we employed dataset GSE24080 to analyze 559 MM patients’ expression profiles. We observed that there was a statistically significant difference of UBE2T expression between three different stages (Fig. 1a, *P* = 1.4e-07, Kruskal–Wallis test). Compared to the expression of UBE2T in stage I MM patients, the expression of UBE2T in stage II and stage III patients showed a significant increase (Fig. 1a, *P* = 0.00015 and *P* = 3e-07, Kruskal–Wallis test). We also compared the expression of UBE2T in different serotype between three stages. In the IgG group, there was a significant difference between different stages (Fig. 1b, *P* = 6.9e-05, Kruskal–Wallis test). It is evident that the expression of UBE2T is increasing with the deterioration of MM. In the IgA and FLC group, the differences of UBE2T expression are also significant (Fig. 1b, *P* = 0.016 and *P* = 0.031, Kruskal–Wallis test). However, UBE2T is more likely to be considered as a meaningful biomarker in an early stage.

### The expression of UBE2T in various molecular types of MM

**Fig. 2:**
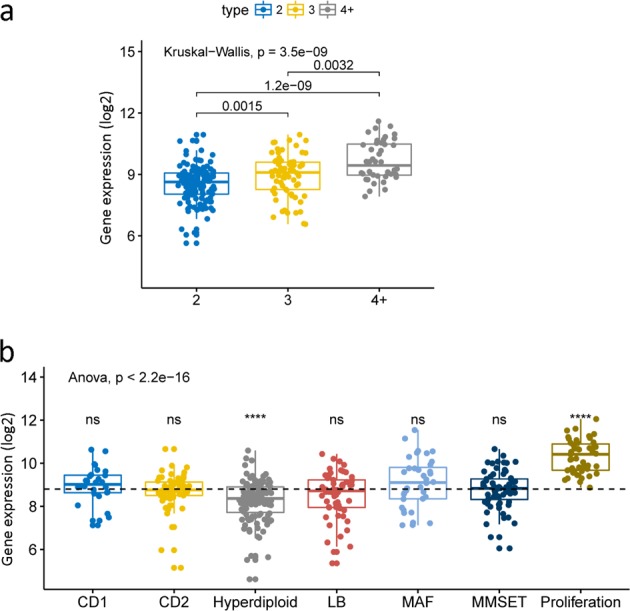
The expression of UBE2T under different amplification of 1q21 and its expression in seven molecular type. **a** The expression of UBE2T increased with the amplification of 1q21. Kruskal–Wallis test. *P* = 3.5e-09. **b** The expression of UBE2T in seven different molecular type MM. Anova analysis test, ns *P* > 0.05, *P ≤ 0.05, **P ≤ 0.01, ***P ≤0.001, ****P ≤ 0.0001

We analyzed the expression of UBE2T under the different amplifications of 1q21. There was a statistically significant difference between different levels of amplifications (Fig. 2a, *P* = 3.5e-09, Kruskal–Wallis test). It is obvious that the expression of UBE2T is increasing with the amplification of 1q21. We also compared the expression of UBE2T in seven different molecular types. UBE2T extremely overexpressed in the proliferation type with a significant difference (Fig. 2b, ****P ≤ 0.0001, Anova analysis test). However, the result in the hyperdiploid type indicated a significant decrease in the level of UBE2T expression (Fig. 2b, ****P ≤ 0.0001, Anova analysis test). In other five types (CD1, CD2, LB, MAF, MMSET), there was no significant difference reported. Additionally, we also analyzed other 308 MM patients in dataset GSE19784. The expressions of UBE2T in nine different MM molecular types were compared in this dataset. The results were consistent with the results in seven molecular types and were statistically significant: UBE2T expression is lower in NFκB type and higher in the proliferation type (Supplemental Fig. 1, *P* = 1.9e-10, Anova analysis test).

### UBE2T gene predicts the survival level in MM

**Fig. 3:**
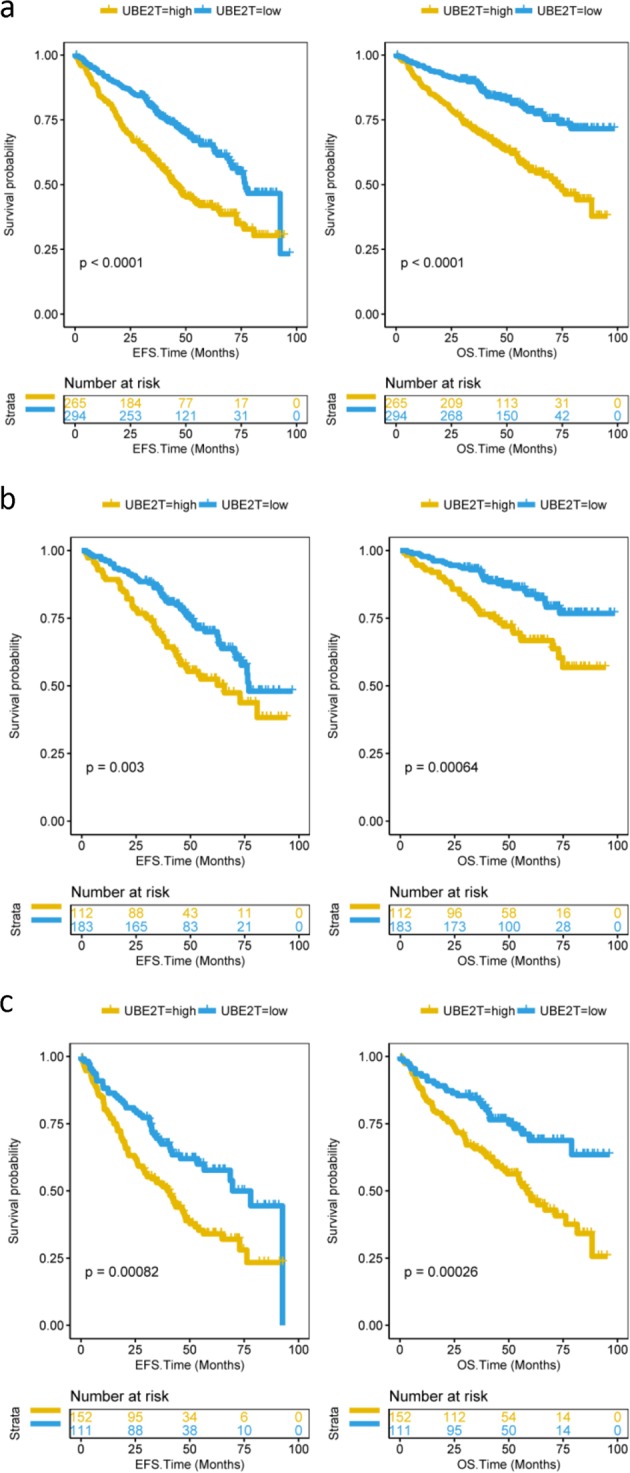
EFS and OS between UBE2T high and low groups. **a** EFS and OS in total 559 MM patients. Compared to the UBE2T low group, the patients in the high group had poor EFS and OS. EFS event-free survival time (months), OS overall survival time (months). Log-rank test. EFS: *P* *<* 0.0001; OS: *P* *<* 0.0001. **b** EFS and OS in stage I MM patients. Compared to the UBE2T low group, stage I patients in the high group had poor EFS and OS. Log-rank test. EFS: *P* = 0.003; OS: *P* = 0.00064. **c** EFS and OS in stage II or stage III patients. Compared to UBE2T low group patients, high group patients had lower EFS and OS. Log-rank test. EFS: *P* = 0.00082; OS: *P* = 0.00026

**Fig. 4:**
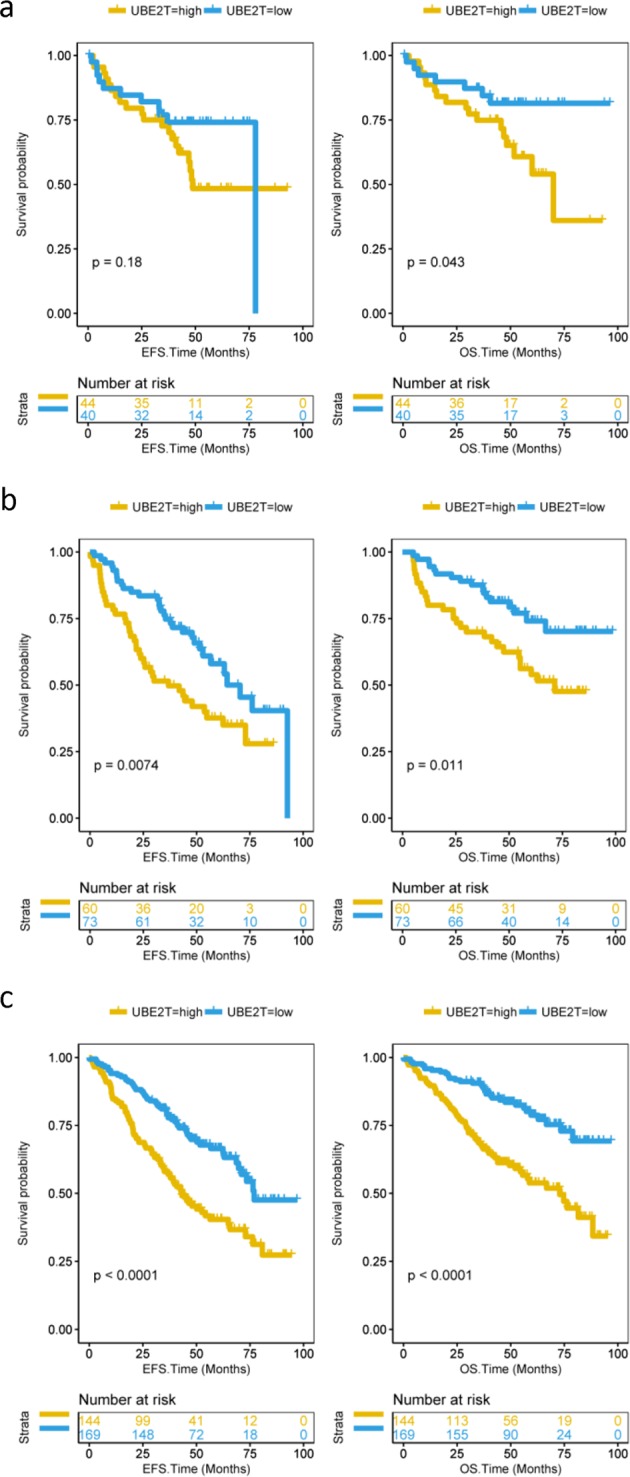
The expression of UBE2T in patients with different serotype MM. **a** EFS and OS between UBE2T high and low groups in patients with FLC serotype MM. Compared to UBE2T low group patients, high group patients had a lower OS. Log-rank test. EFS: *P* = 0.18; OS: *P* = 0.043. **b** EFS and OS between UBE2T high and low groups in patients with IgA serotype MM. Compared to UBE2T low group patients, high group patients had lower EFS and OS. Log-rank test. EFS: *P* = 0.0074; OS: *P* = 0.011. **c** EFS and OS between UBE2T high and low groups in patients with IgG serotype MM. Compared to UBE2T low group patients, high group patients had lower EFS and OS. Log-rank test. EFS: *P* *<* 0.0001; OS: *P* *<* 0.0001

From the previous results, we could assume that overexpression of UBE2T is related to adverse consequences of MM. Thus, we analyzed the prognosis of 559 MM patients in dataset GSE24080. Five hundred and fifty-nine patients were divided into two groups, high expression and low expression groups. We observed that patients in the UBE2T high group had lower both event-free survival (EFS) and overall survival (OS) (Fig. 3a, *P* < 0.0001 and *P* < 0.0001, log-rank test). Besides, we also compared EFS and OS of MM patients in different clinical stages. Compared to the UBE2T low group, high group patients had shorter EFS and OS in stage I (Fig. 3b, *P* = 0.003 and *P* = 0.00064, log-rank test). Consistently, the same results had been showed both in stage II or stage III patients (Fig. 3c, *P* = 0.00082 and *P* = 0.00026, log-rank test). Additionally, the high expression of UBE2T in serotypes IgA and IgG consistently showed significant decrease in EFS and OS (Fig. 4b, *P* = 0.0074 and *P* = 0.011 and Fig. 4c, *P* *<* 0.0001 and *P* *<* 0.0001, log-rank test). In the serotype FLC group, the overexpression of UBE2T is also associated with poor survival. However, it was not statistically significant (Fig. 4a).

### The relationship between UBE2T expression and relapse

**Fig. 5:**
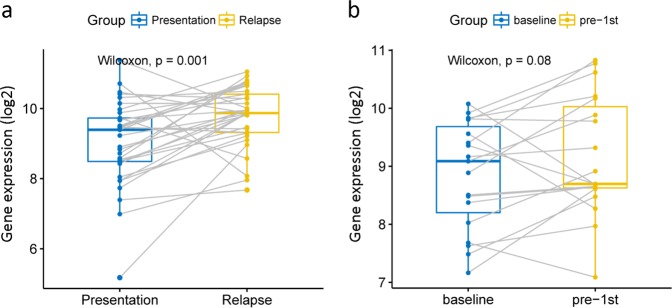
The expression of UBE2T before and after therapy. **a** The expression of UBE2T before and after relapse in 33 MM patients. UBE2T expression was higher after the relapse. Wilcoxon text. *P* = 0.001. **b** The expression of UBE2T at baseline and after the first chemotherapy in 19 MM patients. Wilcoxon text. *P* = 0.08

To investigate the relationship between UBE2T and relapse of MM, we employed two databases GSE83503 and GSE31161 to analyze the expression of UBE2T before and after relapse. There is a statistically significant difference of UBE2T expression between baseline and relapse, and patients who relapsed had a higher UBE2T expression (Supplemental Fig. 2, *P* = 9.2e-05, unpaired *t*-test and Supplemental Fig. 3, *P* = 9.6e-06 and *P* = 0.00055, unpaired *t*-test). We also compared the expression level of UBE2T before and after the relapse in 33 patients. Significant increase of UBE2T was showed after the relapse (Fig. 5a, *P* = 0.001, Wilcoxon test). We also analyzed 19 patients in dataset GSE19554. Compared to the baseline UBE2T, the expression of UBE2T showed a slight increase after the first chemotherapy (Fig. 5b, *P* = 0.08, Wilcoxon test).

### UBE2T is related to cell division

We divided 559 patients into two groups and observed their gene expression profiles of UBE2T between two groups. In all, 181 genes were shown to be up-regulated and 20 down-regulated. In the heatmap, we listed 12 of the most up-regulated and down-regulated genes (Supplemental Fig. 4a, *P* *<* 0.0001, foldchange). In the UBE2T low group, GNG11 was the most down-regulated gene, followed by CTSW and MAP7. While in the UBE2T high group, ASPW was the most up-regulated gene, followed by TOP2A and PHF19 (Supplemental Fig. 4a, *P* < 0.0001, foldchange). The results indicated that overexpression of UBE2T contributes to poor prognosis of MM patients. We also employed Gene Ontology analysis to analyze corresponding pathways. Cell division, mitotic nuclear division, and sister chromatid cohesion are the most three enriched pathways (Supplemental Fig. 4b, *P* *<* 0.0001). We also found that 39 of 40 genes in the cell division pathway were up-regulated, further indicating UBE2T is closely related to the cell proliferation and poor prognosis (Supplemental Fig. 5, *P* *<* 0.0001, unpaired *t*-test).

### The relationship of expression of UBE2T and treatment response

To investigate the relationship between UBE2T expression and treatments' responses, we analyzed 238 patients in database GSE9782 (totally 264 patients). For the bortezomib treatment group, there was no significant difference in UBE2T expression in each treatment responses (left panel of Supplemental Fig. 6, *P* = 0.56, Anova analysis test). For the dexamethasone group, we also found there was no statistical significance (right panel of Supplemental Fig. 6, *P* = 0.41, Anova analysis test). Additionally, we used another database GSE39754. The result showed that there is a significance decrease of UBE2T in the very good partial response (VGPR) group (Supplemental Fig. 7, *P ≤ 0.05, Anova analysis test).

We analyzed UBE2T gene expression in each translocation classification through the GSE9782 dataset. MM were classified into different groups (11q13, 4p16, D1, D1 + D2, D2, D3, MAF) following TC class. UBE2T expression was higher in the D2 group while it was lower in the D1 group (Supplemental Fig. 8, *P* ≤ 0.05, Wilcoxon test). Further, we analyzed UBE2T gene expression and prognosis of MM in GSE9782 dataset. High expression of UBE2T is associated with poor survival and prognosis (Supplemental Fig. 9, EFS: *P* < 0.0001; OS: *P* < 0.0001, log-rank test)

### The expression of UBE2T could be an independent prognostic factor for MM

We employed the Cox regression model to compute multivariate hazard ratios for different variables in dataset GSE24080, totally 559 patients. For EFS, the hazard ratios of magnetic resonance imaging (MRI) (≥3 focal lesions vs <3 focal lesions) and Bone marrow plasma cell (≥ 35% vs <35%) are 1.33 and 1.45 (Supplemental Table 1, *P* = 4.17e-02, *P* = 2.34e-02, Cox regression analysis). Importantly, the hazard ratio of UBE2T (≥8.84 vs <8.84) is 1.69 (Supplemental Table 1, 95% CI: 1.29–2.21, *P* = 1.43e-04, Cox regression analysis). These three factors were significantly related to the EFS in MM patients. For OS, the hazard ratios of B2M (≥3.5 vs <3.5 mg/l) and magnetic resonance imaging (≥3 focal lesions vs <3 focal lesions) are 1.51 and 1.68 (Supplemental Table 1, *P* = 3.73e-02, *P* = 3.14e-03, Cox regression analysis). The hazard ratio of UBE2T is 2.00, indicating close association with OS (Supplemental Table 1, 95% CI: 1.43–2.80, *P* = 5.47e-05, Cox regression analysis). We also analyzed the baseline characteristics between UBE2T high and low groups. There were no significant differences in age, gender, race and isotype between two groups (Supplemental Table 2, *P* *>* 0.05, unpaired *t*-test, two sided). However, the differences in B2M, C-reactive protein, lactate dehydrogenase, albumin, magnetic resonance imaging between two groups showed statistically significant (Supplemental Table 2, *P* ≤ 0.001, unpaired *t*-test, two sided).

## Discussion

MM is a malignant plasma cell tumor with the characteristics of plasmacytosis in the bone marrow and secretion of monoclonal immunoglobulin [27, 28]. In recent years, numerous studies are conducted on MM and significant heterogeneity of MM has been observed in studies [29, 30]. The median overall survival for patients who suffered from MM has improved from 3 to 6 years due to the development of treatments [31]. It is important to improve the outcomes for MM patients. UBE2T is a crucial gene in the FA pathway that is closely associated with DNA repair in vivo [32]. It is reported that UBE2T is closely related to the development and poor prognosis of several cancers such as prostate or gastric cancer [33, 34]. However, limited studies reported the relationship between UBE2T and MM. Therefore, we analyzed several datasets and found out that UBE2T could be considered as an early biomarker for MM and reveal the relationship between UBE2T and the prognosis of MM.

In our study, we analyzed 2684 patients and found UBE2T is a bad indicator of prognosis in MM patients. First, the expression of UBE2T is increasing with the exasperation of MM [35]. Compared with stage I patients, the expression of UBE2T in stage II and stage III patients obviously increased, especially in stage III. Second, compared with the expression in different serotypes of MM, the expression of UBE2T is the most related to IgG type. Consistently, patients who suffered from IgG type MM endure poor diagnosis [36], which indicated high expression of UBE2T is related to a poor outcome. Third, the amplification of 1q21 indicated the poor outcome of MM [37]. Consistently, it is revealed that UBE2T expressed higher with the increasing of amplification. Forth, various molecular types of MM suggested different survival and prognosis for MM. Proliferation is the type with the worst survival and prognosis [22, 38]. However, the expression of UBE2T increased a lot in these two types compared to other types. Fifth, compared with the UBE2T expression high group, patients in the expression low group reported longer EFS and OS. Sixth, patients who suffered from relapse showed higher UBE2T expression.

Biomarkers are important indicators for the diagnosis and treatment of cancers. Therefore, it is of great importance to find out more crucial biomarkers for MM, especially early biomarkers. Early diagnosis of MM contributes to better prognosis and survival for patients [39]. It is necessary to uncover early biomarkers to promote the prognosis of MM patients. In our study, we found UBE2T is a meaningful indicator for MM staging, especially in the early stage. Compared to the expression in stage II and III, UBE2T reported a statistically significant decrease in stage I. It has a close association with the exasperation of MM. However, the increase in the late stage was not significant. Besides, compared to the high UBE2T expression group of stage I patients, the low expression group showed a higher EFS and OS. Therefore, it is more important in the early stage.

From the result of UBE2T expression among seven different molecular types of MM, we observed that the expression of UBE2T in patients with proliferation MM were higher than the mean. Proliferation MM is correlated with poor diagnosis, which further indicated that the expression of UBE2T contributes to poor outcome of MM. UBE2T consistently lower expressed in NFκB type. NFκB signaling is important to the development of MM, activating the noncanonical NFκB pathway and contributing to the prognosis of MM patients [40]. UBE2T is an essential part in the FA pathway that is important to DNA repair. It also has an effect on cell division, therefore, affecting the prognosis of MM. We found that UBE2T is the most related to the cell division pathway and 39 of 40 genes in this pathway were up-regulated. It means that UBE2T contributes to the cell proliferation in the cell cycle, which affects the survival and prognosis of MM.

In recent year, numerous studies focus on the cancer microenvironment and clonal evolution. MM has been considered as a symbol that has been affected by cancer microenvironment and clonal evolution [41]. In our study, we found that UBE2T expressed higher after the first chemotherapy than its baseline expression. There are two probable reasons. First, certain specific factor was triggered by the chemotherapy, increasing the expression of UBE2T. Second, clonal evolution has an impact on UBE2T expression. Low expression clones are likely to be eliminated with the processes of chemotherapy, remaining high expression clones. Therefore, UBE2T showed increase after the first chemotherapy.

Although we employed a large number of samples and followed up for a quite long time, there are still several limitations. In our study, we found that UBE2T could be considered as a meaningful biomarker for MM. However, clinical characteristics and more known biomarkers need to be combined to confirm the impact of UBE2T as the biomarker of MM. Additionally, we observed that UBE2T is likely to affect MM by regulating the cell division pathway; further researches need to be conducted to investigate the mechanism on how UBE2T promotes MM proliferation. In our study, we only postulate UBE2T may be related to clonal evolution of MM; however, more researches such as single-cell sequencing study need to be used to confirm this postulation.

In conclusion, we studied the relationship between UBE2T and MM. UBE2T is a predictor of MM survival, higher the expression of UBE2T, worse the prognosis and survival of MM patients. UBE2T increases with the exasperation of MM. UBE2T are probably affecting MM through the cell division pathway, which could be a meaningful biomarker for MM.

## Supplementary information

Supplemental Table

Supplemental Figure

Supplemental legends
