# Supplementary material for: High expression of UBE2T predicts poor prognosis and survival in multiple myeloma
Source: Cancer Gene Ther. 2019 Jan 9;26(11):347–55. doi: 10.1038/s41417-018-0070-x (PMC6892417; doi:10.1038/s41417-018-0070-x)
Supplement: Supplementary file 1 — Supplemental Table [file 41417_2018_70_MOESM1_ESM.docx]

Table S1. Multivariate analysis of clinical prognostic parameters in 559 multiple myeloma patients (Cox regression multivariate analysis).

|  |  | 95% CI for HR | |  |
| --- | --- | --- | --- | --- |
|  | HR | Lower | Upper | *P*-value |
| EFS |  |  |  |  |
| B2M (>= 3.5 mg/l) | 1.34 | 0.99 | 1.84 | 6.20e-02 |
| ALB (>= 35 g/l) | 0.86 | 0.61 | 1.22 | 3.98e-01 |
| HGB (>= 100 g/l) | 0.80 | 0.59 | 1.08 | 1.41e-01 |
| MRI (>= 3 focal lesions) | 1.33 | 1.01 | 1.75 | 4.17e-02 |
| BMPC (>= 35%) | 1.45 | 1.05 | 1.99 | 2.34e-02 |
| UBE2T (>=8.84) | 1.69 | 1.29 | 2.21 | 1.43e-04 |
|  |  |  |  |  |
| OS |  |  |  |  |
| B2M (>= 3.5 mg/l) | 1.51 | 1.02 | 2.21 | 3.73e-02 |
| ALB (>= 35 g/l) | 0.72 | 0.49 | 1.07 | 1.04e-01 |
| HGB (>= 100 g/l) | 0.89 | 0.62 | 1.28 | 5.30e-01 |
| MRI (>= 3 focal lesions) | 1.68 | 1.19 | 2.37 | 3.14e-03 |
| BMPC (>= 35%) | 1.39 | 0.93 | 2.07 | 1.05e-01 |
| UBE2T (>=8.84) | 2.00 | 1.43 | 2.80 | 5.47e-05 |

Beta-2 microglobulin (B2M), mg/l; Albumin (ALB), g/l; Haemoglobin (HGB), g/l; Number of magnetic resonance imaging (MRI)-defined focal lesions (skull, spine, pelvis); Bone marrow biopsy plasma cells (BMPC, %); Event-free survival time (EFS, months), determined from date of registration to the occurrence of death from any cause, disease progression or relapse, or censored at the date of last contact; Overall survival time (OS, months), determined from date of registration to the date of death from any cause or censored at the date of last contact. HR, hazard ratio; CI, confidence interval.

Table S2. Baseline patient characteristics according to the expression level of UBE2T.

|  |  | UBE2T-low | UBE2T-high | *P*-value |
| --- | --- | --- | --- | --- |
| n |  | 294 | 265 |  |
| AGE (mean (sd)) |  | 56.54 (9.37) | 57.89 (9.53) | 0.092 |
| SEX (%) | female | 107 (36.4) | 115 (43.4) | 0.109 |
|  | male | 187 (63.6) | 150 (56.6) |  |
| RACE (%) | other | 37 (12.6) | 25 ( 9.4) | 0.294 |
|  | white | 257 (87.4) | 240 (90.6) |  |
| ISOTYPE (%) | FLC | 40 (14.0) | 44 (17.3) | 0.321 |
|  | IgA | 73 (25.6) | 60 (23.6) |  |
|  | IgD | 0 ( 0.0) | 3 ( 1.2) |  |
|  | IgG | 169 (59.3) | 144 (56.7) |  |
|  | Nonsecretory | 3 ( 1.1) | 3 ( 1.2) |  |
| B2M (mean (sd)) |  | 3.96 (4.27) | 5.59 (6.27) | <0.001 |
| CRP (mean (sd)) |  | 8.58 (16.83) | 14.99 (27.93) | 0.001 |
| CREAT (mean (sd)) |  | 1.19 (1.00) | 1.47 (1.51) | 0.01 |
| LDH (mean (sd)) |  | 157.64 (51.35) | 187.88 (76.02) | <0.001 |
| ALB (mean (sd)) |  | 4.14 (0.52) | 3.95 (0.63) | <0.001 |
| HGB (mean (sd)) |  | 11.46 (1.79) | 11.02 (1.82) | 0.005 |
| ASPC (mean (sd)) |  | 40.75 (22.71) | 44.84 (25.90) | 0.053 |
| BMPC (mean (sd)) |  | 44.55 (25.51) | 48.46 (27.01) | 0.084 |
| MRI (mean (sd)) |  | 8.21 (12.90) | 14.23 (15.61) | <0.001 |
| Cytogenetic abnormality (%) | No | 208 (70.7) | 144 (54.3) | <0.001 |
|  | Yes | 86 (29.3) | 121 (45.7) |  |
| Subgroup (%) | CD1 | 10 ( 3.4) | 18 ( 6.9) | <0.001 |
|  | CD2 | 30 (10.3) | 28 (10.7) |  |
|  | HY | 82 (28.2) | 33 (12.6) |  |
|  | LB | 32 (11.0) | 26 ( 9.9) |  |
|  | MF | 16 ( 5.5) | 20 ( 7.6) |  |
|  | MS | 33 (11.3) | 33 (12.6) |  |
|  | MY | 88 (30.2) | 57 (21.8) |  |
|  | PR | 0 ( 0.0) | 47 (17.9) |  |
| Therapy (%) | TT2 | 172 (58.5) | 173 (65.3) | 0.119 |
|  | TT3 | 122 (41.5) | 92 (34.7) |  |

n, number of patients; C-reactive protein (CRP), mg/l; Creatinine (CREAT), mg/dl; Aspirate plasma cells (ASPC, %).
