# Supplementary material for: High expression of UBE2T predicts poor prognosis and survival in multiple myeloma
Source: Cancer Gene Ther. 2019 Jan 9;26(11):347–55. doi: 10.1038/s41417-018-0070-x (PMC6892417; doi:10.1038/s41417-018-0070-x)

Supplementary Figure 1

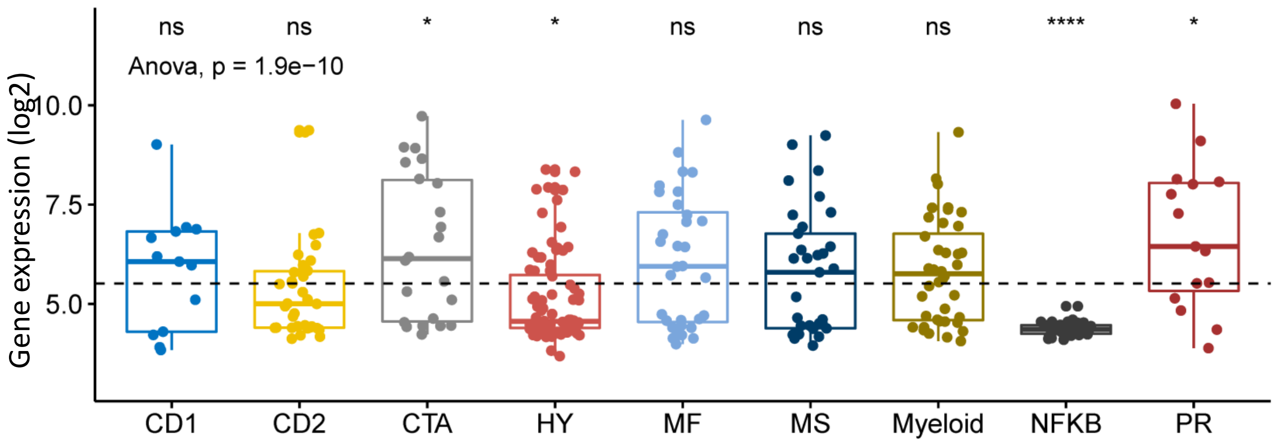

Supplementary Figure 2

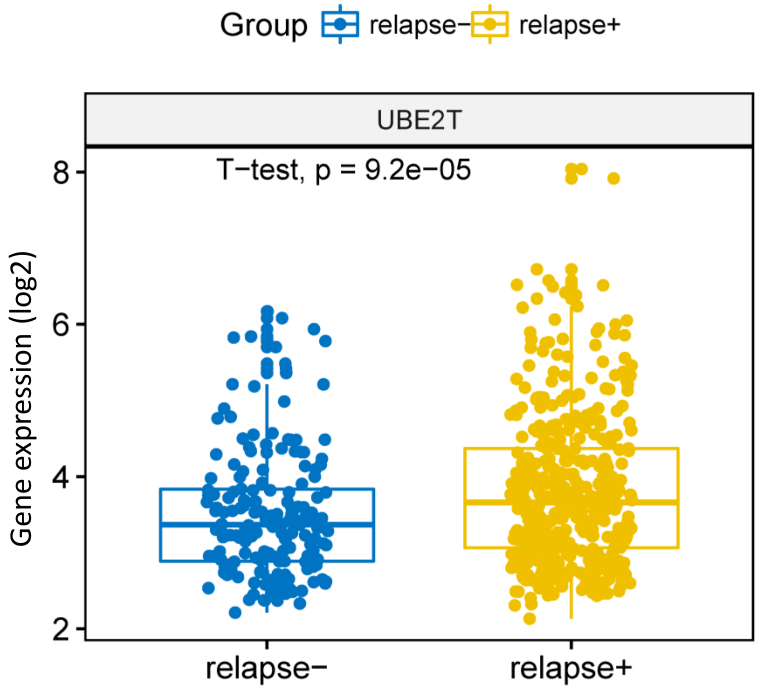

Supplementary Figure 3

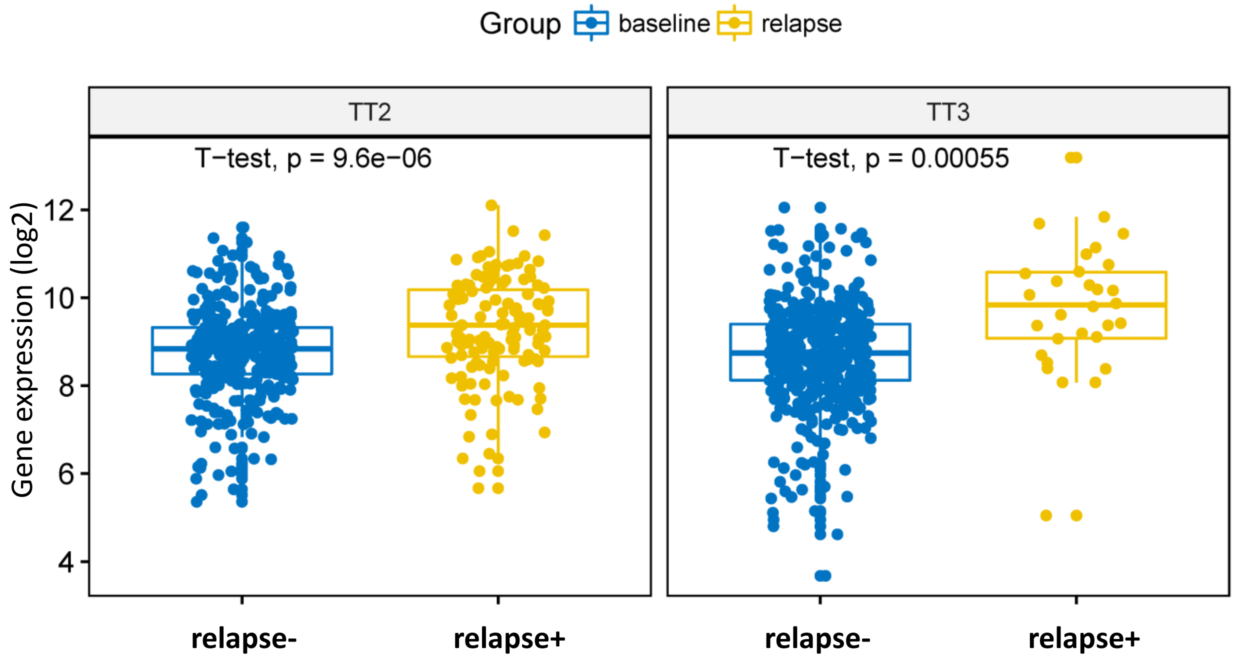

Supplementary Figure 4

a

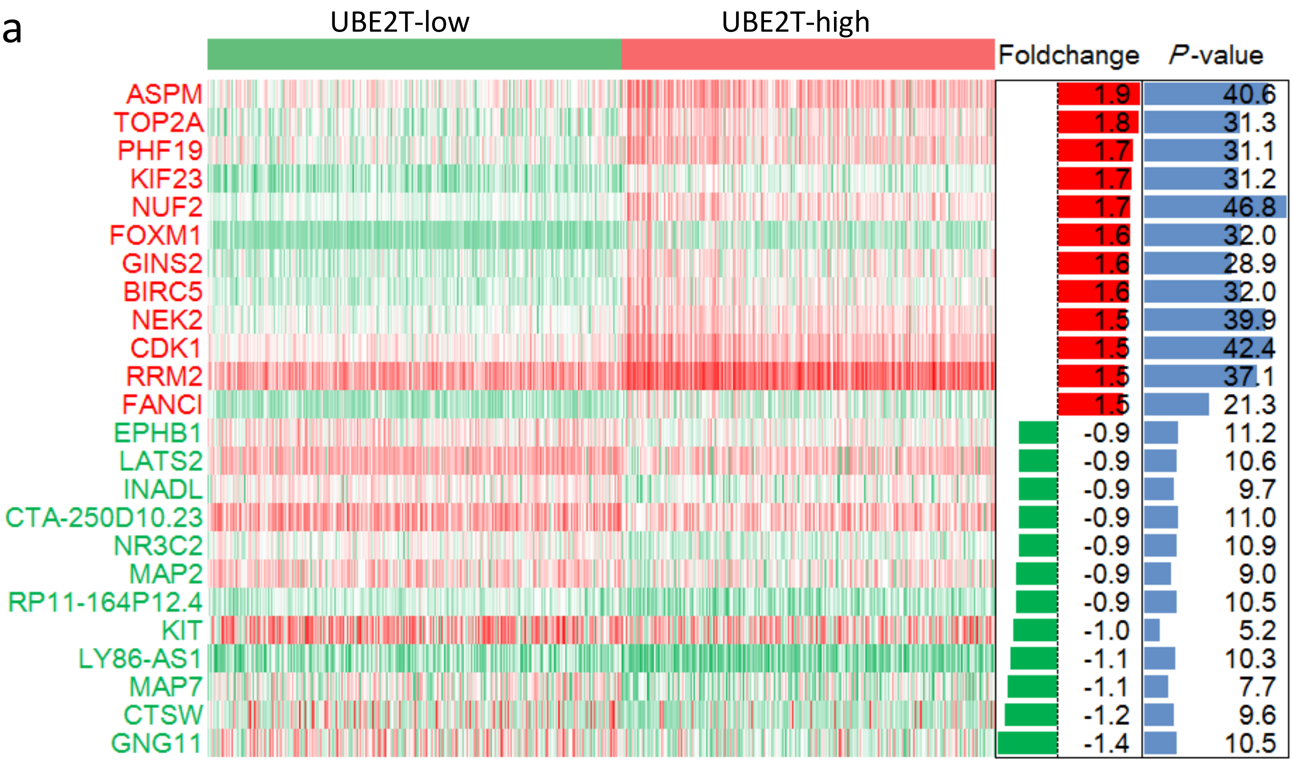

b

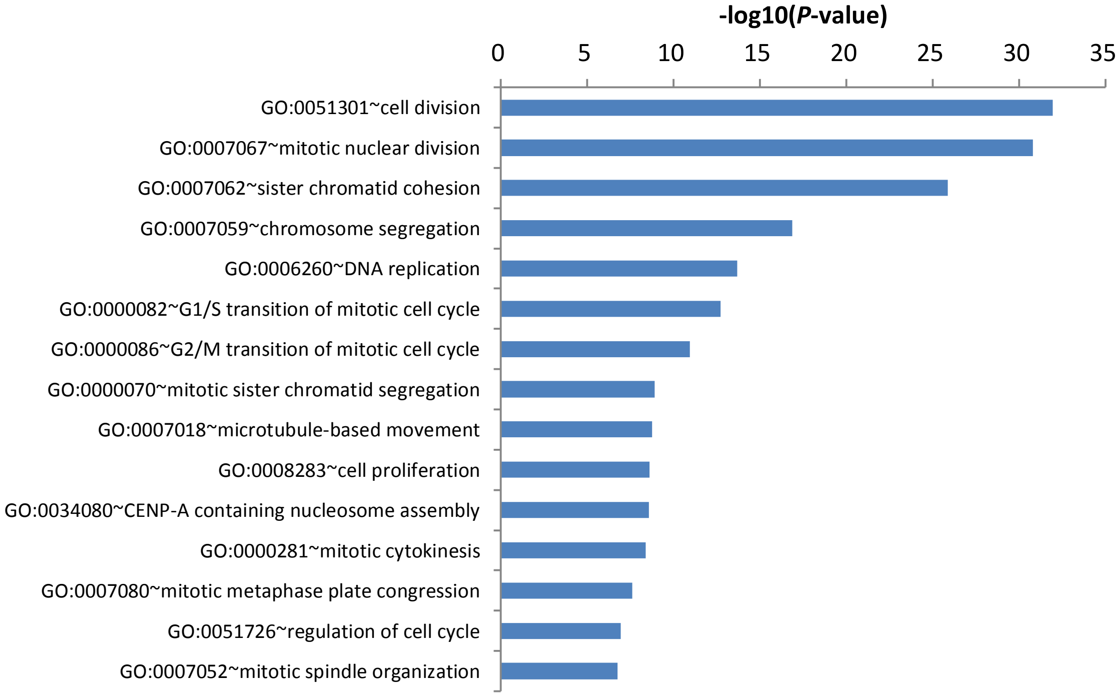

Supplementary Figure 5

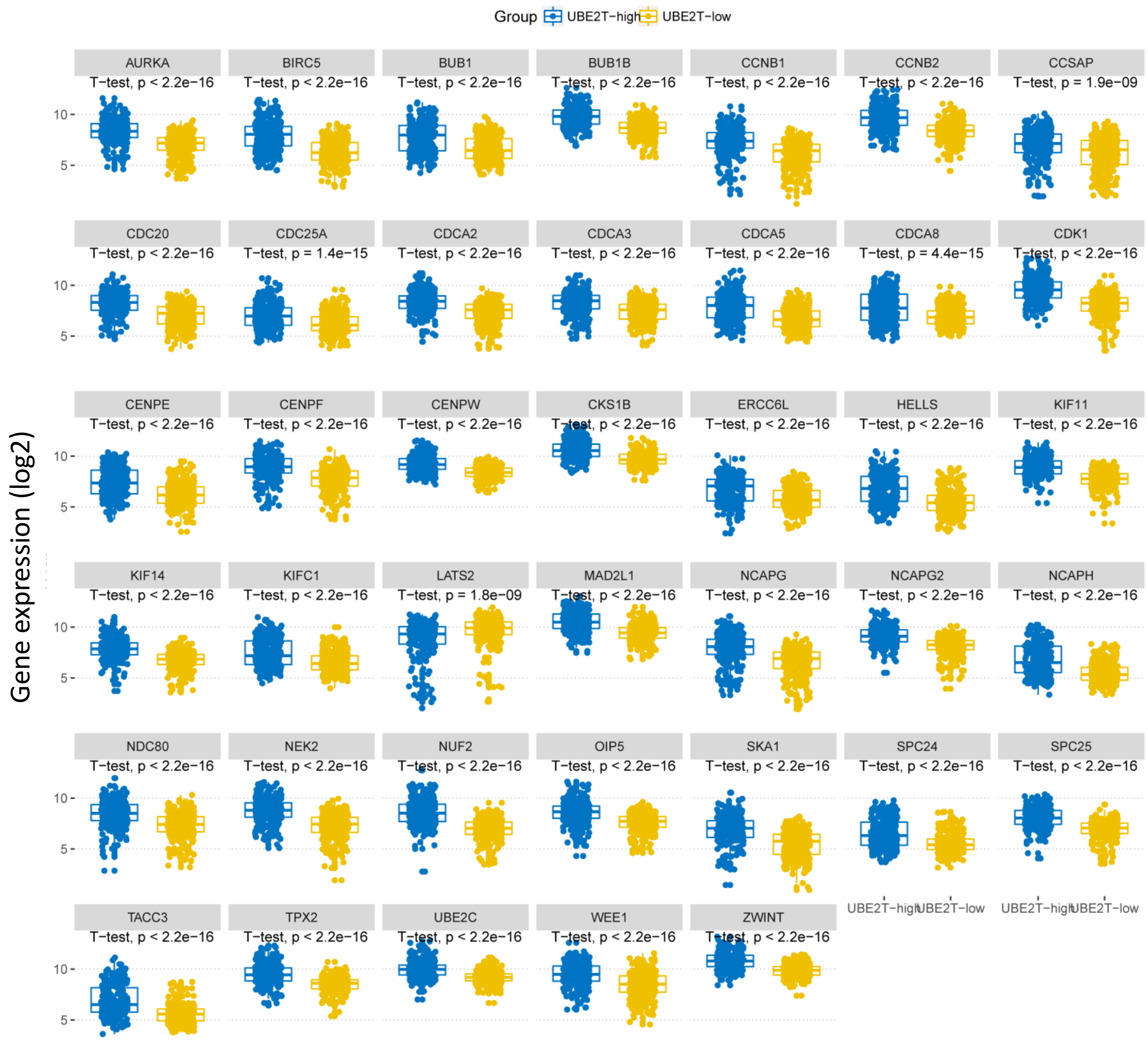

Supplementary Figure 6

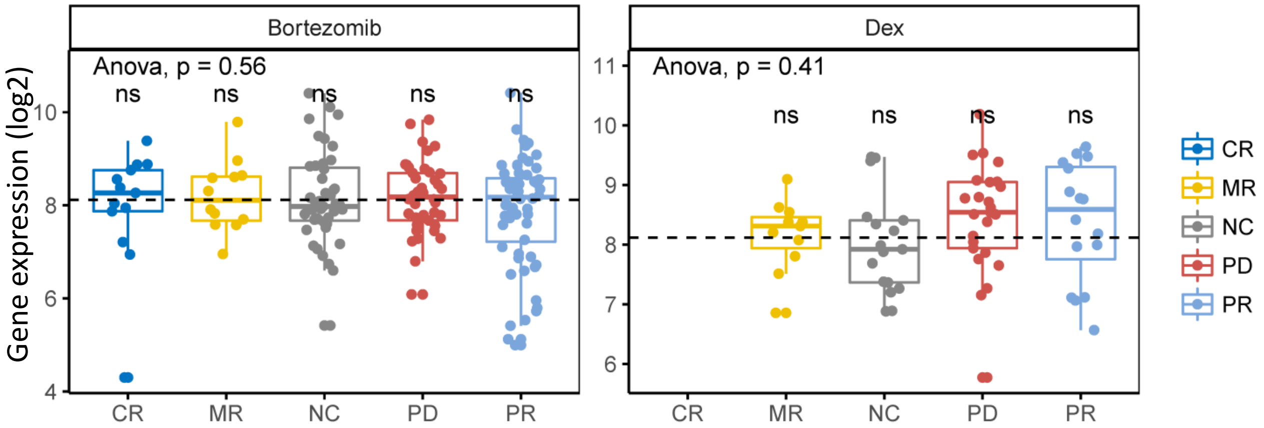

Supplementary Figure 7

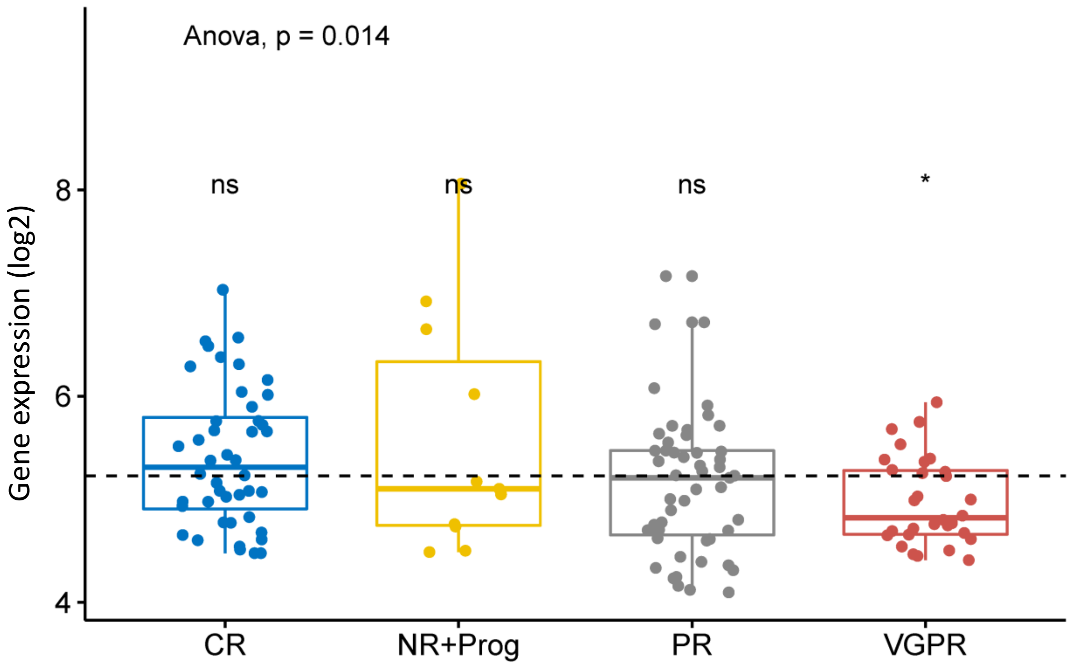

Supplementary Figure 8

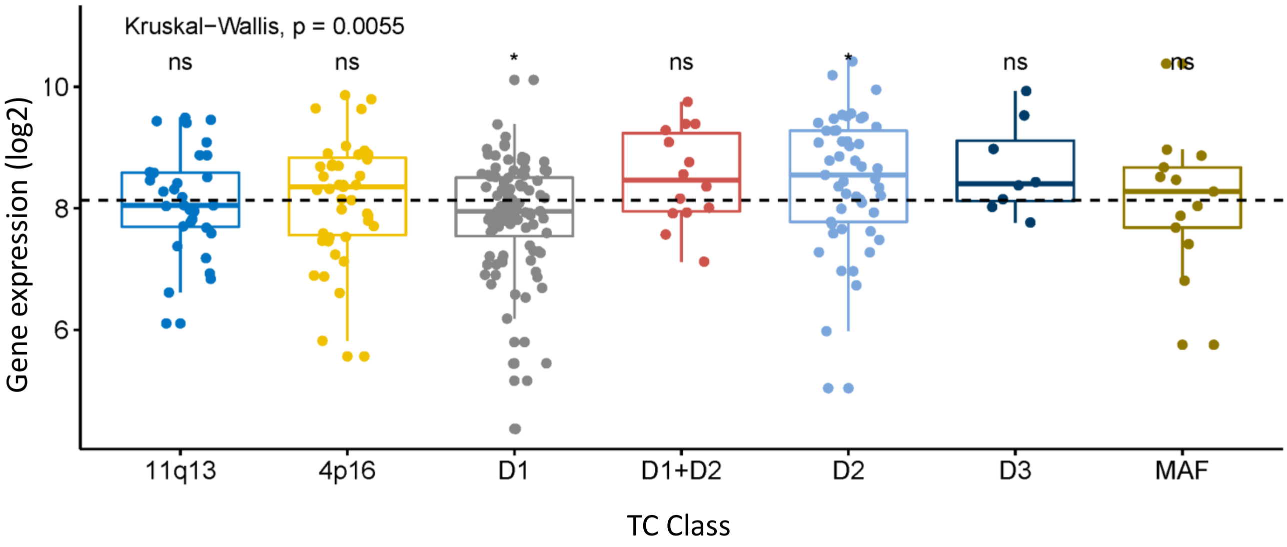

Supplementary Figure 9

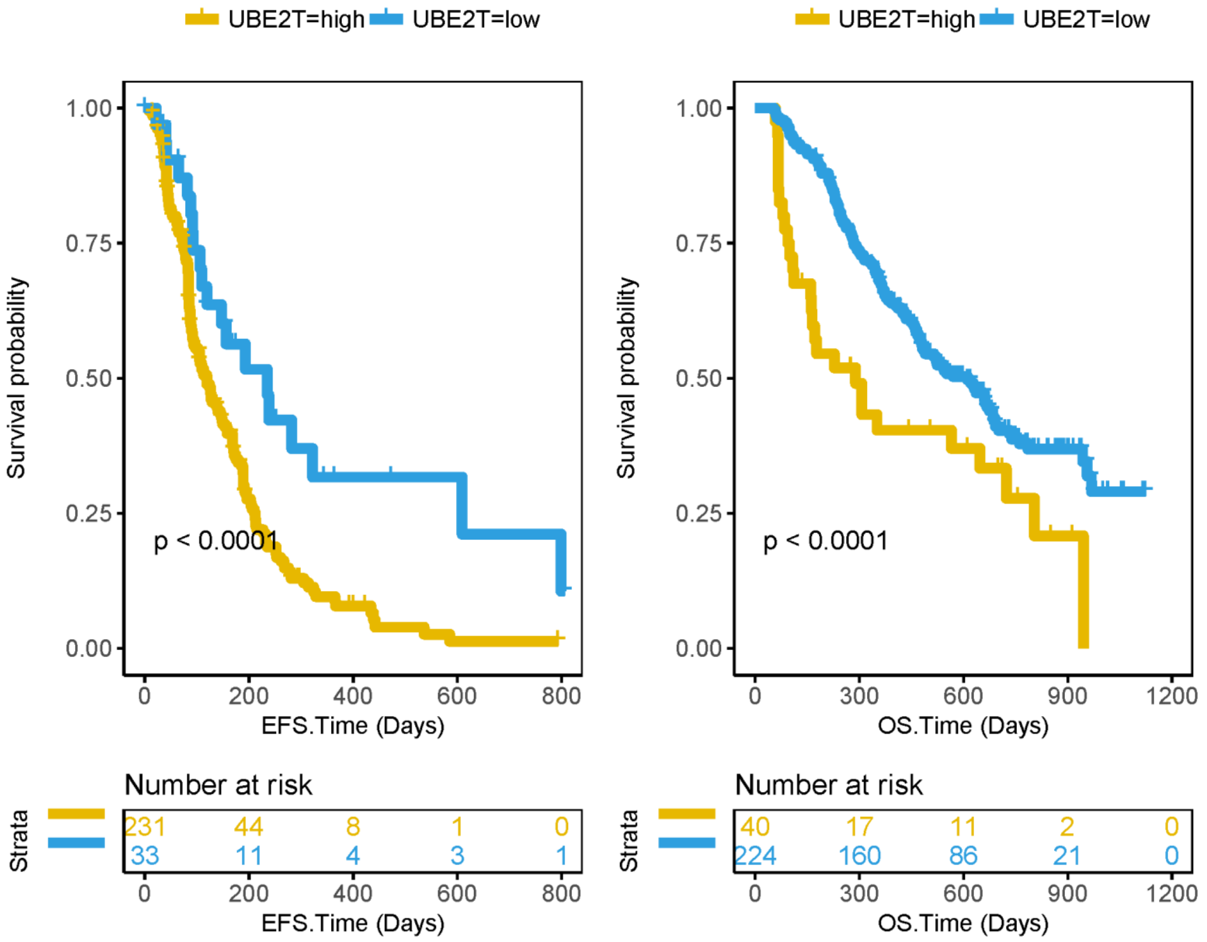

Supplement: Supplementary file 2 — Supplemental Figure [file 41417_2018_70_MOESM2_ESM.pdf]
