# Supplementary material for: High expression of UBE2T predicts poor prognosis and survival in multiple myeloma
Source: Cancer Gene Ther. 2019 Jan 9;26(11):347–55. doi: 10.1038/s41417-018-0070-x (PMC6892417; doi:10.1038/s41417-018-0070-x)
Supplement: Supplementary file 3 — Supplemental legends [file 41417_2018_70_MOESM3_ESM.docx]

**Supplemental Fig. 1** The expression of UBE2T in 9 different molecular types of MM. In HY and NFKB type, UBE2T showed a significant decrease. In CTA type, the expression of UBE2T were increased. Anova analysis test, ns: *P* > 0.05, *: *P* <= 0.05, **: *P* <= 0.01, ***: *P* <= 0.001, ****: *P* <= 0.0001.

**Supplemental Fig. 2** The expression of UBE2T between relapse patients and non-relapse patients.

The expression of UBE2T in relapse patients showed a significant increase. unpaired t-test. *P*= 9.2e-05.

**Supplemental Fig. 3** The expression of UBE2T between relapse and non-relapse patients with TT2 and TT3 treatments. UBE2T showed a significant increase after the relapse in both TT2 and TT3 patients. Total therapy 2 (TT2); Total therapy 3 (TT3). TT2: unpaired t test. *P* = 9.6e-06. TT3: unpaired t test. *P* = 0.00055.

**Supplemental Fig. 4** Differential gene expressions between UBE2T high and low group.

a Different gene expression between UBE2T high and low group. The heatmap showed 12 the most up-regulated and down-regulated genes. Red implied high expression, green implied low expression. The right side are related foldchange (log2) and *P*-value (-log10).

b Top 15 of the most enriched pathway for different UBE2T expression in MM patients. X-axis: *P*-value (-log10).

**Supplemental Fig. 5** In the cell division pathway, different gene expression between UBE2T high and low group. unpaired t test.

**Supplemental Fig. 6** The expression of UBE2T to the different treatment response.

No significant difference of UBE2T expression in both bortezomib and dexamethasone group. Anova analysis test. Bortezomib: *P* = 0.56. Dexamethasone: *P* = 0.41.

**Supplemental Fig. 7** The expression of UBE2T to the triple drug therapy response.

The expression of UBE2T showed a significant decrease in VGPR group. Triple drug therapy – VAD: Vincristine, Adriamycin, and Dexamethasone - as induction therapy followed by Autologous Stem Cell Transplant (ASCT) as a maintenance therapy. 'Treatment response' was measured after ASCT. Treatment responses: Complete Response (CR); Partial Response (PR); Very Good Partial Response (VGPR); No Response, Stable disease (NR); No Response, Progressive disease (Prog). Anova analysis test. *P* = 0.014.

**Supplemental Fig. 8** UBE2T gene expression in each translocation classification in the GSE9782 dataset.

MM were classified into different groups (11q13, 4p16, D1, D1+D2, D2, D3, MAF) following TC class. UBE2T high express in D2 group while low express in D1 group. Kruskal-Wallis test, ns: *P* > 0.05, *: *P* <= 0.05.

**Supplemental Fig. 9** EFS and OS between UBE2T high and low group in GSE9782 dataset.

EFS and OS in total 264 MM patients in GSE9782 dataset. Compared to UBE2T low group, the patients in high group had a poor EFS and OS. EFS, Event-free survival time (Days); OS, Overall survival time (Days). Log-rank test. EFS: P < 0.0001; OS: P < 0.0001.
